# The Negative Impact of COVID-19 on Life Insurers

**DOI:** 10.3389/fpubh.2021.756977

**Published:** 2021-09-27

**Authors:** Xun Zhang, Pu Liao, Xiaohua Chen

**Affiliations:** ^1^China Institute for Actuarial Science/School of Insurance, Central University of Finance and Economics, Beijing, China; ^2^School of Finance, Jiangxi University of Finance and Economics, Nanchang, China

**Keywords:** COVID-19, mortality rates, risk, life insurer, financial sustainability

## Abstract

Understanding COVID-19 induced mortality risk is significant for life insurers to better analyze their financial sustainability after the outbreak of COVID-19. To capture the mortality effect caused by COVID-19 among all ages, this study proposes a temporary adverse mortality jump model to describe the dynamics of mortality in a post-COVID-19 pandemic world based on the weekly death numbers from 2015 to 2021 in the United States. As a comparative study, the Lee-Carter model is used as the base case to represent the dynamics of mortality without COVID-19. Then we compare the force of mortality, the survival probability and the liability of a life insurer by considering COVID-19 and those without COVID-19. We show that a life insurer's financial sustainability will deteriorate because of the higher mortality rates than expected in the wake of COVID-19. Our results remain unchanged when we also consider the effect of interest rate risk by adopting the Vasicek and CIR models.

## Introduction

The COVID-19 pandemic has posed a significant challenge to the operation of the insurance industry around the world. Due to COVID-19, life insurers in Australia suffered a net loss of $1.8 billion for the year ending March 2020, compared with a profit of $759 million in the previous year (1). The pandemic also causes great damage on the U.S. life insurance industry. For instance, Prudential reported a $2.41–billion net loss in the second quarter of 2020 as opposed to a $738–million net income in the second quarter of 2019 (2). Accordingly, rating agencies revised their outlook from stable to negative for life insurers worldwide. In particular, AM Best changed its outlook for the U.S. life insurance industry from stable to negative (3). One factor behind these changes in the life sector's outlook is: the possibility of higher mortality rates than anticipated. To address this crucial and timely issue caused by COVID-19, it is significant for life insurers to adopt an appropriate mortality model by including COVID-19 mortality risk to analyze their financial sustainability.

The COVID-19 pandemic has adversely affected mortality rates, increasing the mortality risk of life insurers. Understanding the adverse effect of COVID-19 on mortality rates is very important because higher mortality rates than expected will affect the future financial sustainability process of life insurers. Consequently, life insurers must incorporate COVID-19 mortality risk to better forecast their financial sustainability based on an appropriate mortality model. As the objective of this study, we provide life insurers in the U.S. with a temporary adverse mortality jump model by including the effect of COVID-19 on mortality rates to enable better analysis of financial sustainability during the pandemic. To produce such a model, the direct and indirect effects of the pandemic on mortality rates must first be understood. The direct effect reflects only the death numbers directly caused by COVID-19. However, the under-diagnosis, limited COVID-19 testing and imperfect test sensitivity in early 2020 resulted in undercounting of the number of deaths due to COVID-19 (4–6). Underestimation of the deaths caused by an emergency is common. For example, only 31 official deaths due to Chikungunya virus were recorded between 2014 and 2015 in Puerto Rico, whereas 1,310 excess deaths were estimated in a time-series analysis (7). Therefore, the additional deaths caused by the indirect effects of COVID-19, i.e., the potential consequences associated with the pandemic, should not be ignored. Government interventions imposed during the pandemic may indirectly affect other causes of deaths (8). For instance, many people with serious existing illnesses were unable to seek timely medical treatment after the outbreak of COVID-19, thus influencing their physical and mental health and decreasing their life expectancy (9–11). Many people lost their jobs or income in this tough period due to the self-isolation policies proposed by the government or the economic recession caused by COVID-19; consequently, suicide rates increased because of the long-term unemployment (12–16). Crowded emergency departments were also responsible for additional deaths (17, 18). Furthermore, even people recovering from COVID-19 may have temporary or long-term kidney and liver failure, thus decreasing the survival probability among many recovered patients (19, 20). The limited access to health services, physical and psychological effects of social distancing, and economic changes have had indirect and adverse effects on mortality rates. Death rates are always higher than expected during emergencies. According to Weinberger et al. (2020)'s test, the number of excess deaths was 28% higher than the official COVID-19-reported death numbers from March 1^*st*^ to May 30^*th*^, 2020 (21). Given that the indirect effects of COVID-19 may be much greater than the direct effect, we use the excess death numbers to explain the adverse mortality jump caused by this pandemic rather than using the official COVID-19 death counts (21, 22). The method of computing the excess deaths has also been used for other pathogens, such as pandemic influenza viruses and HIV (23–25).

In this study, we aim to compare the difference between the liability of the whole life insurance during the COVID-19 pandemic (2020 and 2021) and pre-COVID-19 period (2015–2019) to show the negative effect of COVID-19 on life insurer's financial sustainability. Therefore, life insurers' financial sustainability can be viewed as a function of random mortality rates. Several dynamic mortality models have sought to describe a three-dimensional surface of mortality by modeling mortality trends involving both age-dependent and time-dependent terms (26–36). However, very few studies have considered mortality jumps (37–40). Our model is inspired by this stream of research. We first use the well-known Lee-Carter model (1992) as the basis to calculate the pre-COVID-19 force of mortality without including information on the Spanish flu in 1918 (26). We choose the force of mortality to describe the mortality change because its evolution can describe the cohort effect in the general trend of the mortality (37). Then we adjust the model of Cox et al. (37) to capture the adverse mortality effect caused by COVID-19. We highlight that the adverse effect on mortality rates during the COVID-19 pandemic should be temporary based on the historical data of pandemics such as the 1918 flu pandemic. Moreover, COVID-19 vaccination in 2021 suggests a potential for mortality to rapidly return to baseline levels (41). We calibrate our mortality model based on the up-to-date weekly deaths data from the U.S. Centers for Disease Control and Prevention (CDC). Our results show that the financial sustainability of a life insurer typically deteriorates during the COVID-19 pandemic. Given this, we should forecast future mortality rates by incorporating the negative effect induced by COVID-19. In addition to the mortality rates, the financial sustainability of life insurers also depends on the discount rates, which are correlated with the interest rate risk. Accordingly, we also forecast the financial sustainability of life insurers during the pandemic by adding the interest rate risk based on the Vasicek and CIR models (42, 43).

We contribute to the literature in three aspects. First, our temporary adverse mortality jump model has the advantages of capturing the COVID-induced mortality risk and incorporating the framework of the well-known Lee-Carter model. Second, this paper investigates the effect of COVID-19 on the mortality rates among different ages and provides some illustrative results for the liability and financial sustainability of a life insurer over a long-term (30 years) horizon. Third, we also consider the term structure of interest rates (e.g., the Vasicek and CIR models) to study the effect of interest rate risk on a life insurer's financial sustainability during the COVID-19 pandemic.

The remainder of this paper is organized as follows. Mortality Risk introduces the stochastic mortality models used to analyze the adverse mortality effect caused by COVID-19. We then describe the framework of the whole life insurance. Numerical Illustration provides a numerical example illustrating the difference in the force of mortality, k-year survival rates and the financial sustainability of a life insurer during the COVID-19 pandemic and pre-COVID-19 period. Additional Analysis: Interest Rate Risk shows the effect of interest rate risk on a life insurer's financial sustainability. Finally, Conclusions presents the conclusion.

## Mortality Risk

Practitioners usually calculate the present value of a life insurance company by using fixed mortality rates. However, this deterministic approach underestimates the uncertainties of mortality rates. Therefore, we use the stochastic mortality models in this article to capture the randomness of mortality rates. Mortality modeling can use different types of mortality rates because one type of mortality rates is correlated with others. In this section, we use the force of mortality μ(*x, t*) to develop two different mortality models. We further assume that the force of mortality remains the same in each age interval [*x, x*+1]. Hence, for age *x*+*s*, at time *x*+*s*, we have μ(*x*+*s, t*+*s*) = μ(*x, t*), where 0 ≤ *s* < 1.

### Mortality Risk Without COVID-19

In this section, we adopt the well-known Lee-Cater mortality model to describe the mortality dynamics of the insured without COVID-19, which can be expressed as follows:


(1) $$\text{ln }\mu \left(x,t\right)=a\left(x\right)+b\left(x\right)k\left(t\right)+\;\varepsilon_{x,t},$$

where **ε**_***x*,*t***_ is an error term with mean 0 and variance $\sigma_{\varepsilon }^{2}$, which explains the historical influences not captured by this model. ***ln*μ**(***x*,*t***) is the logarithm of the force of mortality for age x in year t. The parameters ***a*(*x*)** and ***b*(*x*)** are age-specific constants, where ***a*(*x*)** is the age group shift effect, and ***b*(*x*)** indicates the response at each age to the mortality time-series ***k*(*t*)**. The time-varying mortality index ***k*(*t*)** represents general mortality trends, which can be modeled as a random walk with a drift g:


(2) $$k\left(t-1\right)=k\left(t-1\right)+g+e_{t},\;e_{t}\sim \;N\left(0,\;\sigma_{k}^{2}\right),$$

where *e*_*t*_ is the error term with mean 0 and variance $\sigma_{k}^{2}$. On the basis of the estimated *k*(*t*) from singular value decomposition (SVD), the parameters *g* and $\sigma_{k}^{\;}$ in (2) can be easily calibrated with standard statistical techniques. Of note, the drift parameter *g* is a negative value, which suggests an improvement in mortality rates. With the estimated â(*x*_0_+*t*), $\hat{b}\left(x_{0}+t\right)$ and $\tilde{k}\left(t\right)$, the future force rate of mortality $\hat{\mu }\left(x_{0}+t\right)$ for age *x*_0_+*t* at time *t* can be expressed as follows:


(3) $$\tilde{\mu }\left(x_{0}+t,t\right)=e^{\hat{a}\left(x_{0}+t\right)+\hat{b}\left(x_{0}+t\right)\tilde{k}\left(t\right)},\;t=1,2,\;\ldots$$

where $\tilde{k}\left(t\right)$ can be forecasted by the time series model (2) with the estimated *g* and σ_*k*_. Finally, the forecasted one-year survival probability ${\tilde{p}}_{x_{0}+t,t}$ at different ages and years is equal to:


(4) $${\tilde{p}}_{x_{0}+t,t}=1-{\tilde{q}}_{x_{0}+t,t}=e^{-\tilde{\mu }\left(x_{0}+t,t\right)},t=1,2,\ldots ,$$

where ${\tilde{q}}_{x_{0}+t,t}$ denote the forecasted probability that the insured aged *x*_0_+*t* at time *t* dies before reaching age *x*_0_+*t*+ 1.

### Mortality Risk Including COVID-19

To capture the adverse mortality effect caused by the pandemic, we adjust the model of Cox et al. (37) to explain the excess death rates caused by COVID-19. The force of mortality after the outbreak of the pandemic μ_*J*_(*x, t*) is given by:


(5) $$\mu_{J}\left(x,t\right)=\;\mu \left(x,t\right)*e^{H\left(x,t\right)},$$

where μ(*x, t*) is the force of mortality of age x at time t predicted from equation (3). H(x, t) is the mortality jump process caused by the pandemic, which is equal to:


(6) $$H\left(x,t\right)=\;\sum_{j=1}^{N(t)}\;e_{j}\;C_{j}\left(x\right)\;exp\left(-\kappa_{j}\left(t-\tau_{j}\right)\right)1_{\left\{t\leq \tau_{j}\right\}},$$

where jump event *j* counts the number of the Poisson process *N*(*t*) with an arrival rate of λ_τ_ by time *t*. τ_*j*_ is the time at which pandemic event *j* occurs. The factor (*t*−τ_*j*_) provides the cumulative mortality deterioration as *t* increases after time τ_*j*_ of the jump event *j*. Function 1_{*t*≥_τ__*j*_}_ is equal to 1 if the jump event *j* occurs and zero otherwise. The parameter *e*_*j*_ is the maximum severity of the adverse mortality jump event *j* among all ages. *C*_*j*_(*x*) is an age-specific function measuring the effect of jump event *j* on the mortality of people at age x with respect to the maximum severity *e*_*j*_. Therefore, *C*_*j*_(*x*) ranges from 0 to 1. *C*_*j*_(*x*) = 0 indicates that the jump event *j* has no effect on the mortality rate at age *x*, whereas *C*_*j*_(*x*) = 1 indicates that people at age x experience the most severe effect due to the pandemic *j*. The parameter κ_*j*_ represents the time period of the pandemic's effect on mortality rates: the time span of jump event *j* increases (decreases) as κ_*j*_ decreases (increases). For simplicity, we further assume that each jump event *j* has the same effect on mortality rates. Accordingly, we have *e*_*j*_ = *e*, *C*_*j*_(*x*) = *C*(*x*) and κ_*j*_ = κ. Then equation (6) can be rewritten as follows:


(7) $$\begin{array}{r} H\left(x,t\right)=\;\overset{N\left(t\right)}{\underset{j=1}{\sum }}\;e_{\;}\;C_{\;}\left(x\right)\;exp\left(-\kappa_{\;}\left(t-\tau_{j}\right)\right)1_{\left\{t\geq \tau_{j}\right\}}. \end{array}$$

After calibrating Lee and Carter's model without the pandemic event, and using the mortality data correlated with COVID-19 from CDC to estimate the parameter *e* and function *C*(*x*), we forecast the force of mortality based on the COVID-19 pandemic $\tilde{\mu_{J}\;}\left(x_{0}+t\right)$ with a determined pandemic arrival rate λ_τ_ and pandemic duration κ. Similarly, the forecasted one-year survival probability with the adverse mortality effect caused by COVID-19 $\tilde{p_{J}}{\;}_{x_{0}+t,t}$ at different ages and years equals:


(8) $${\tilde{p_{J}}}_{x_{0}+t,t}=1-{\tilde{q_{J}}}_{x_{0}+t,t}=t=1,2,...$$

where $\tilde{q_{J}}{\;}_{x_{0}+t,t}$ denote the forecasted probability that the insured aged *x*_0_+*t* at time *t* dies before reaching age *x*_0_+*t*+1 during the COVID-19 pandemic.

### Whole Life Insurance

In this section, we use the liability of the whole life insurance as an example to aid in analysis of a life insurer's financial sustainability. Suppose a life insurer underwrites whole life insurance policies to *N*_0_(*x*_0_) participants, where *x*_0_ is the age when the policy issued. In this life insurance contract, if the insured dies, he or she will receive a benefit of *E* at the end of the year; that is, if the insured survives, there are no death payments are made at the end of each year. Given *N*_0_(*x*_0_+*t*) dead insured people at age *x*_0_+*t* at time t, the life insurer's liability *L*(*t*) at time *t* is


(9) $$L\left(t\right)=N_{0}\left(x_{0}+t\right)*E^{*}a_{x_{0}+t},\;t=0,\;1,\;2,\;\ldots$$

where *a*_*x*_0_+*t*_ is the conditional expected value of the life insurance for age *x* = *x*_0_+*t* at time *t*. The present value of the life insurance *a*_*x*_0_+*t*_ can be viewed as a special portfolio of zero coupon bonds with stochastic coupon payment and different maturities:


(10) $$a_{x}=a_{x_{0}+t}=\sum_{k=1}^{\infty }v_{tk}^{k{\bar{p}}_{x,t}{\tilde{q}}_{x,t}}.$$

The variable ${}_{k}^{\;}{\bar{p}}_{x,t}$ is the conditional expected *k*-year survival rates for age *x* at time *t*, which is a random variable and can be expressed as follows:


(11) $$k{\bar{p}}_{x,t}=\tilde{p}x,t*{\tilde{p}}_{x+1,t+1}*{\tilde{p}}_{x+2,t+2}\ldots^{*}{\tilde{p}}_{x+k-1,t+k-1}.$$

Amid COVID-19, formulae (9) and (11) can also be used to compute the liability *L*_*J*_(*t*) and the k-year survival rates ${}_{k}^{\;}{\bar{p_{j}}}_{\;x,t}\;$associated with COVID-19, respectively. The one-year discount factor *v*_*t*_ in (10) is equal to


(12) $$\begin{array}{r} v_{t}=\frac{1}{1+r_{L,t}}, \end{array}$$

where *r*_*L, t*_ represents the discount rate at time *t*. In (10), the conditional expected value _*a*_*x*_0+*t*_ depends on two variables: the conditional expected k-year survival rate ${}_{k}^{\;}{\bar{p}}_{x,t}$ and the one-year discount factor *v*_*t*_. To model the liability of the life insurer in the distant future, we must consider the random feature of the mortality rates and interest rates. The mortality rates and interest rates are independent even in the real world; thus, we further assume independence between the mortality rates and interest rates in this article. To show the adverse effect of COVID-19 on the financial sustainability of a life insurer, we only consider the mortality risk in Numerical Illustration. In Additional Analysis: Interest Rate Risk, we will consider both mortality risk and interest risk.

## Numerical Illustration

**Figure 1:**
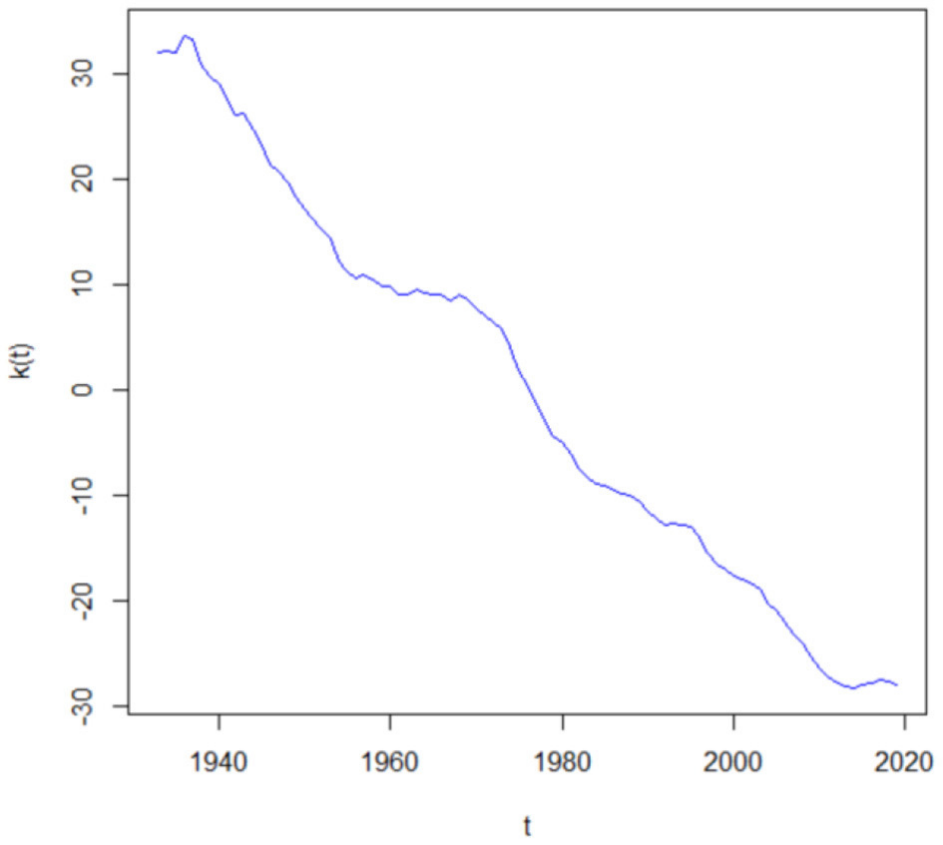
Estimated *k*(*t*) from *t* = 1933 to *t* = 2019.

In this section, we use an example to show the difference in the force of mortality, the *k*-year survival probability and the liability of the life insurer during the COVID-19 pandemic and pre-COVID-19 period. We assume that a U.S. life insurer has a number of *N*_0_(35) = 10, 000 life insurance policies sold at time 2020, and the benefit payment at the end of each year starting from *t* = 2021 is *E* = 1. Furthermore, the mortality dynamics of the insured are assumed to be the same as the U.S. total (both male and female) population. Then, on the basis of the U.S. population mortality table for both sexes from age 35 to 105 in the Human Mortality Database from 1933 to 2019, we calibrate the Lee-Carter mortality model through SVD. Figure 1 represents the mortality index values *k*(*t*). The decreasing trend in the mortality index *k*(*t*) indicates the improvement of mortality rates along the time. We then use these estimated *k*(*t*) to estimate *g* = −0.3214 and σ_*k*_ = 0.3590 in equation (2).

**Figure 2:**
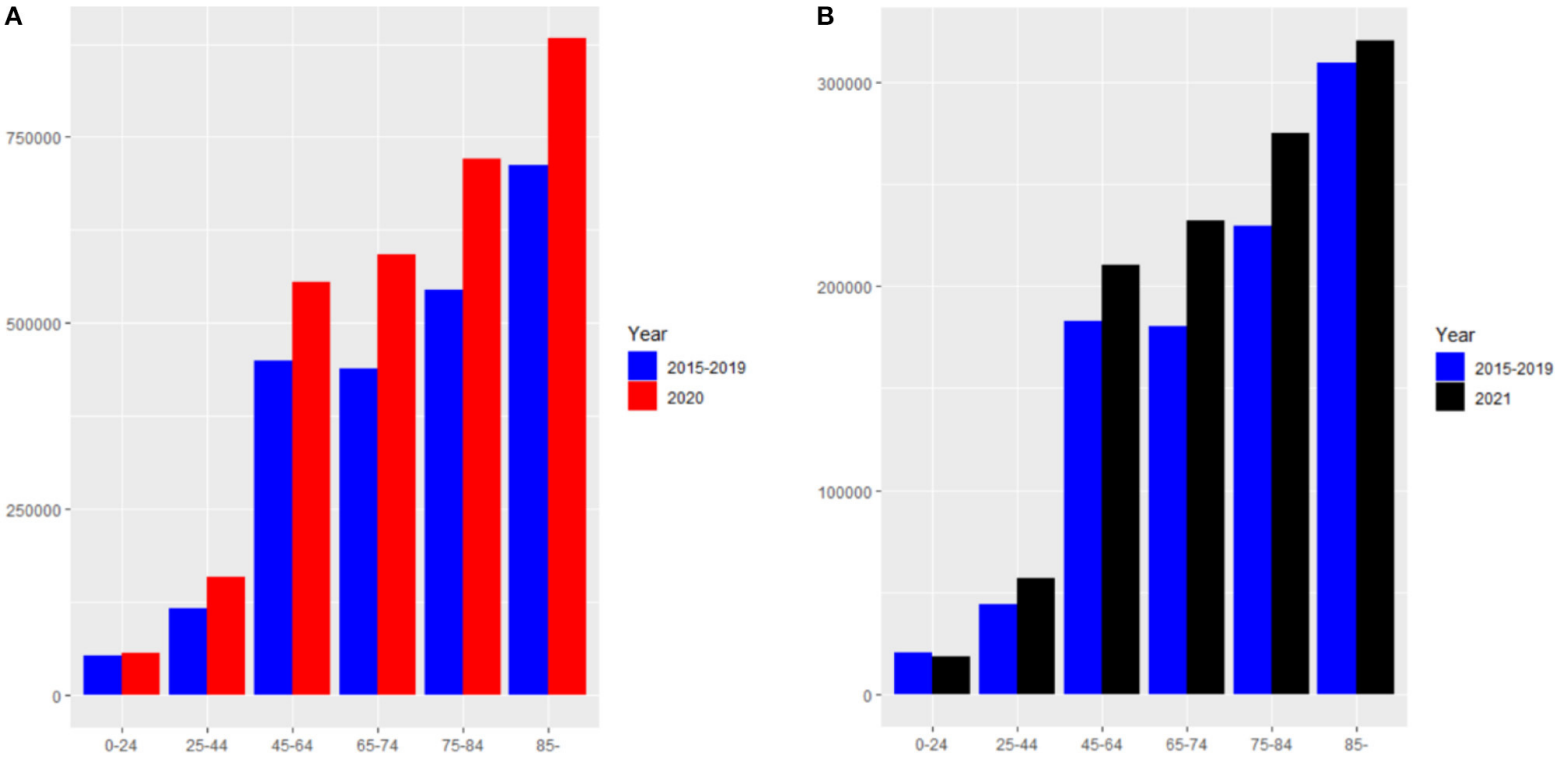
**(A)** The death numbers from the 9^*th*^ week to the 52^*nd*^ week in 2020 and the average death numbers in the same period from 2015 to 2019. **(B)** The death numbers from the 1^*st*^ week to the 17^*th*^ week in 2021 and the average death numbers in the same period from 2015 to 2019.

This paper focuses on the overall (i.e., both direct and indirect) effects of the COVID-19 pandemic on mortality rates. One way of monitoring the excess death rates is to compare the number of deaths after the outbreak of COVID-19 with the number of deaths before COVID-19. On the basis of the weekly deaths in the U.S. provided by the CDC from January 2015 to May 2021, we first compute the excess death numbers of each age group in 2020 by comparing the death numbers from the 9^*th*^ week to the 52^*nd*^ week in 2020 with the average death numbers in the same period from 2015 to 2019. The excess number of deaths in 2021 can also be obtained by comparing the death numbers within the 17^*th*^ week with the average number of deaths in the same period from 2015 to 2019. The age groups in our data include: under 25 years, 25–44 years, 45–64 years, 65–74 years, 75–84 years, as well as 85 years or older. The results of excess death numbers correlated with COVID-19 for each age group are shown in Figure 2. In Figure 2A, the blue histograms denote the average deaths between 2015 and 2019 from the 9^*th*^ week to the 52^*nd*^ week, and the red histograms denote the deaths in 2020 in the same period. The excess deaths of 0–24 years, 25–44 years, 45–64 years, 65–74 years, 75–84 years and 85 years or older are 2,980, 42,267, 105,455, 152,510, 175,379, and 170,409, respectively, thus indicating that COVID-19 had the greatest impact on people older than 65 years old in the United States in 2020. The U.S. government has had better control of COVID-19 in 2021, so the excess death numbers have substantially decreased in this year. Specifically, the excess death numbers for each group are −1,742, 13,178, 27,318, 51,415, 45,604 and 10,960, respectively. Of note, the excess death number for people younger than 25 years is negative in 2021, which suggests an improvement of mortality along the time. Therefore, we assume that the excess death rate of age under 25 is 0 in 2021.

With the observed excess deaths, the excess death rate of each age group can be computed by a simple equation:


(13) $$\begin{array}{r} excess\;death\;rate_{2020,2021}=\frac{excess\;deaths_{2020,2021}}{the\;average\;deaths_{2015-2019}\;}. \end{array}$$

The results of the excess death rate for each age group are shown in Table 1, which provides further support for COVID-19 having more serious impact on mortality rates in 2020 than 2021 in the United States. For example, COVID-19 has had only a small effect on people 85 years or older in 2021, as compared with 2020, because the government has taken better measures to protect the elderly from the effects of this pandemic, and people also have a better understanding of the transmission mechanisms of COVID-19. Consequently, the effects of COVID-19 will be much smaller with good compliance with stringent distancing measures, high treatment rates and health-system capacity (44).

**Table 1:** Excess death rate for each age group in 2020 and 2021.

**Age group**	**2020**	**2021**
under 25 years	0.0559	0
25–44 years	0.3649	0.3001
45–64 years	0.2345	0.1494
65–74 years	0.3469	0.2848
75–84 years	0.3218	0.1987
85 years or older	0.2391	0.0354

**Figure 3:**
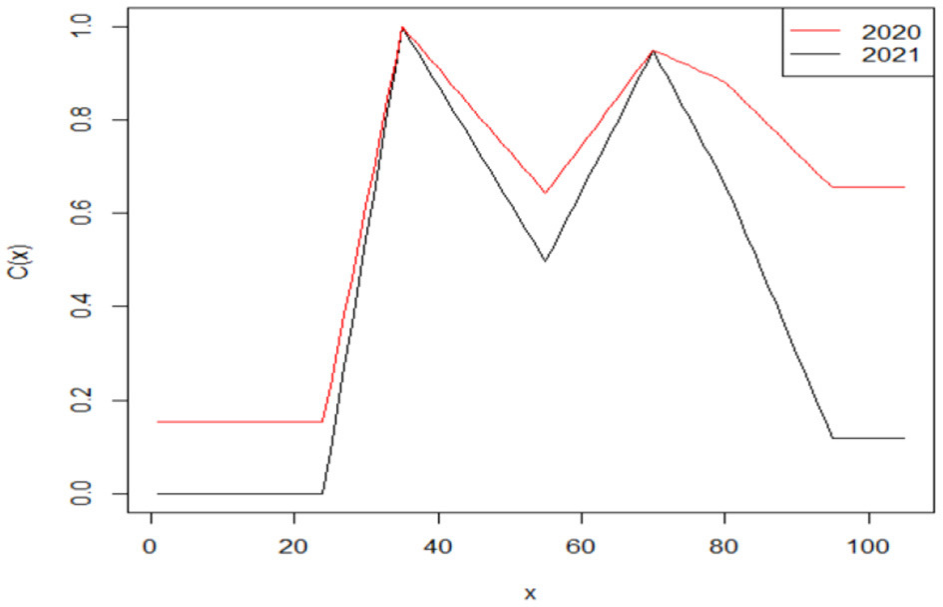
Adverse mortality effect of COVID-19 on $\tilde{\mu_{\;}}\left(x_{0}+t,t\right)$ across ages.

To obtain the excess death rate of each age, we further assume that the excess death rates are the same for people under 25 and over 95 years of age. For any other ages, the excess death rates are assumed to be the excess death rate of the median age of each age group. Therefore, the excess death rates of each age after the COVID-19 pandemic can be computed by the assumption of linear interpolation, which can be expressed as follows:


(14) $$f\left(x\right)=\{\begin{array}{c} d_{1},\;x\leq \;24\\ d_{1}+\frac{x-24}{35-24}\;\left(d_{2}-d_{1}\right),\;25<\;x\leq \;35\;\\ d_{2}+\frac{x-35}{55-35}\;\left(d_{3}-d_{2}\right),\;35<\;x\leq \;55\\ d_{3}+\frac{x-55}{70-55}\;\left(d_{4}-d_{3}\right),\;55<\;x\leq \;70\\ d_{4}+\frac{x-70}{80-70}\;\left(d_{5}-d_{4}\right),\;70<\;x\leq \;80\\ d_{5}+\frac{x-35}{55-35}\;\left(d_{6}-d_{5}\right),\;80<\;x\leq \;95\\ d_{6},\;x\;\leq \;95\; \end{array}\;,$$

where *d*_*i*_, *i* = 1, 2, ⋯ , 6 denotes the excess death rate of each age group from under 25 years (*i* = 1) to 95 years or older (*i* = 6). On the basis of the linear interpolation assumption, our results indicate that people at age 35 experienced the highest excess death rate in both 2020 (*e*_2020_ = 0.3649, *C*_2020_(35) = 1) and 2021 (*e*_2021_ = 0.3001, *C*_2021_(35) = 1). Then we can derive the age-specific function $C{\left(x\right)}_{2020,2021}=\frac{f{\left(x\right)}_{2020,2021}}{e_{2020,2021}}$ for each year. Our results are shown in Figure 3.

**Figure 4:**
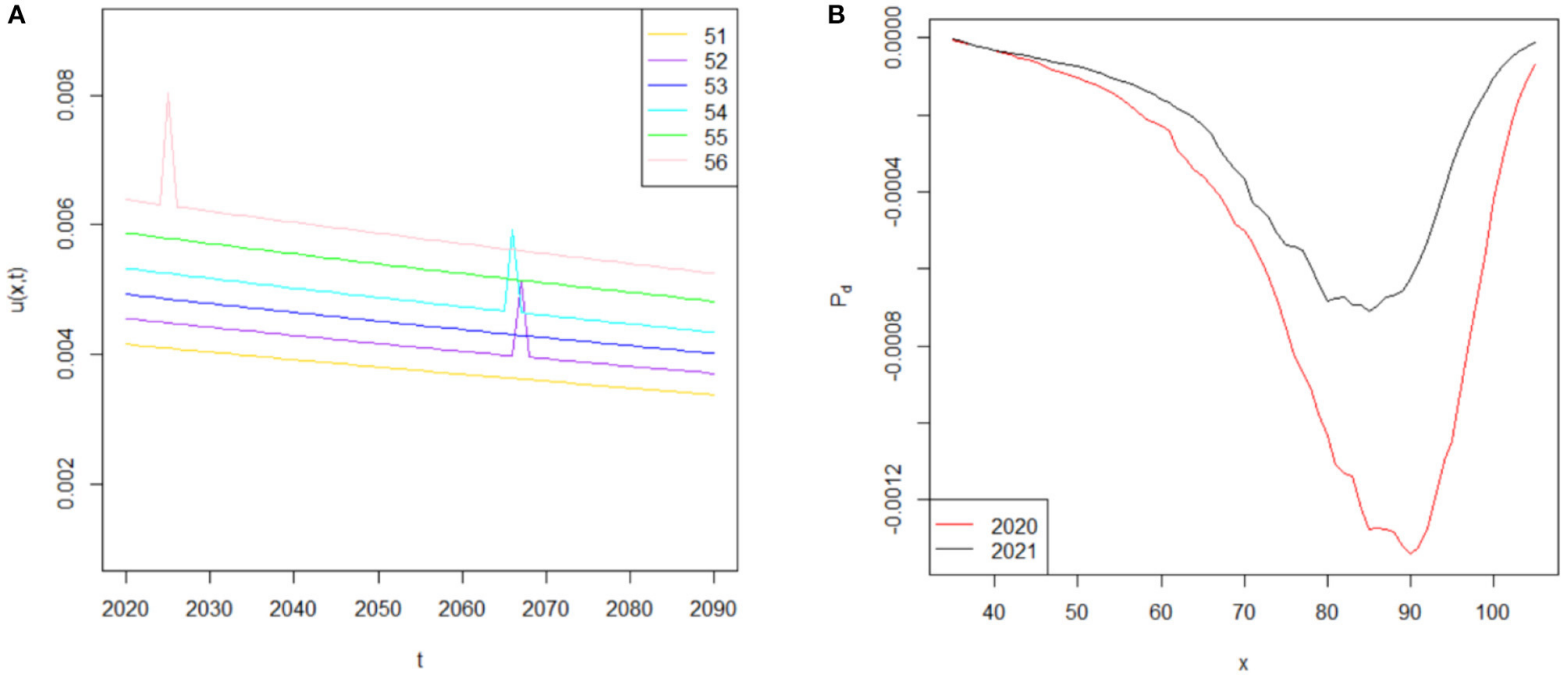
**(A)** Simulated force of mortality from $\tilde{\mu_{J}}\left(51,t\right)$ to $\tilde{\mu_{J}}\left(56,t\right)$. **(B)** The difference in conditional expected k-year survival rate for age 35 at time *t* during the COVID-19 pandemic and pre-COVID-19 period.

On the basis of the age-specific function *C*(*x*), we derive the future force of mortality $\tilde{\mu_{J}}\left(x_{0}+t,t\right)$ for age *x*_0_+*t* at time *t* by assuming the value of arrival rate λ_τ_ = 0.01 and the value of time period κ = 1 of the jump event *j*. Since jump events such as the 1918 flu occur only approximately once per 100 years, and pandemics only last only a few years, the assumptions of λ_τ_ = 0.01 and κ = 1 are reasonable (37). Given this, evolutions of the force of mortality in one simulation iteration from 2020 to 2090 between ages 51 and 56 are shown in Figure 4. The decreasing trend of the force of mortality indicates the improvement of mortality over time as described previously. Specifically, the purple, cyan and pink curves represent the force of mortality following the COVID-19 pandemic. A sudden surge in the force of mortality occurs for several years when a jump event *j* occurs, but then the force of mortality rapidly decreases to normal levels, owing to the hypothesis that the pandemic lasts only a short period. If a jump event does not occur in the future, then the force of mortality based on the COVID-19 pandemic will be equal to that in Lee-Carter's model $\tilde{\mu_{J}}\left(x_{0}+t,t\right)=\tilde{\mu_{\;}}\left(x_{0}+t,t\right)$, as shown by the gold, blue and green curves. To further highlight the difference between these two mortality models, we also compare the conditional expected *k*-year survival rate for age 35 at time *t* during the COVID-19 pandemic (i.e., 2020 and 2021) and those before COVID-19 (i.e., 2015–2019) in Figure 4B. On the basis of the average of the 1,000 simulation paths for the k-year survival probability ${}_{k}^{\;}{\bar{p}}_{x,t}$ and ${}_{k}^{\;}{\bar{p_{j}}}_{\;x,t}$, we compute the average difference between these two survival rates $P_{d}=\;\bar{{}_{k}^{\;}{\bar{p_{j}}}_{\;x,t}}-\bar{{}_{k}^{\;}{\bar{p}}_{x,t}}\;<0$, which indicates that the higher COVID-19 force of mortality induces a lower survival rate. Our results in Figure 4B show that COVID-19 produces a more serious effect on the conditional expected *k*-year survival probability in 2020 (red curve) than in 2021 (black curve). For example, the conditional expected *k*-year survival probability for age 35 will decrease by a maximum of 0.0013 in 2020, which is almost two times higher than that in 2021 (0.0007). Moreover, on the basis of the deaths data in 2020, the adverse mortality impact caused by COVID-19 will continue to increase (decrease) for a 35-year-old individual who lives before (after) the age of 90, which indicates the importance of adopting an appropriate mortality model for life insurers after the outbreak of COVID-19.

**Figure 5:**
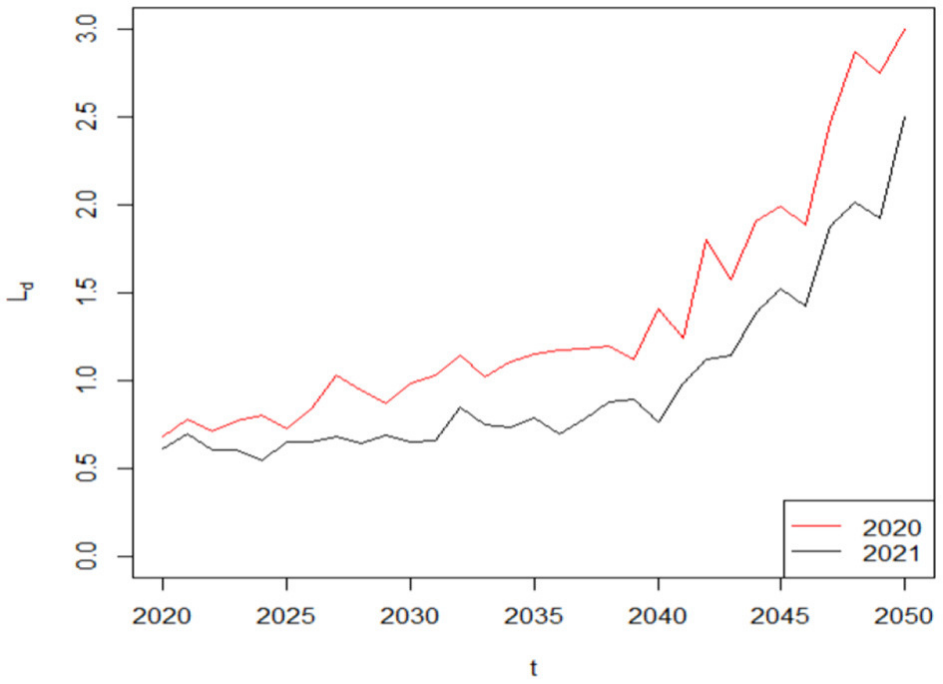
The liability difference in 2020 and 2021 with COVID-19 induced mortality risk.

The financial sustainability of a life insurance company usually improves (deteriorates) as liability decrease (increase). To analyze the difference in the liability of the life insurer over the decision period of *T* = 30 based on the COVID-19 induced mortality risk, we assume that the term structure of interest rate is flat: *r*_*L, t*_ = *r* = 0.05. Given that the insured will receive the benefit payment as long as he or she is dead at year *t*, we hypothesize the lower survival probability caused by COVID-19 will induce higher average benefit payments and so do the average liability. Figure 5 supports our hypothesis, where $L_{d}=\bar{L_{J}\left(t\right)}-\bar{L_{\;}\left(t\right)}>0$. For simplicity, the average difference in the liability of the life insurer during the COVID-19 pandemic and pre-COVID-19 period *L*_*d*_ is called the liability difference hereafter. In our example, the liability difference *L*_*d*_ is in million dollars. As time passes, the number of survivors in this life insurance policy gradually declines, so the liability difference *L*_*d*_ in 2020 gradually increases from 0.68 (0.61) million dollars to 3.00 (2.50) million dollars in 2050 based on the 2020 (2021) weekly deaths data from CDC. Therefore, COVID-19 has a negative and growing effect on a life insurer's financial sustainability, which indicates that mortality models without considering the impact of COVID-19 on life insurers cannot be applied in a post-COVID-19 world. Furthermore, our results suggest that the liability of the life insurer in 2020 is higher than that in 2021, which provides further support to the hypothesis that COVID-19 had posed more adverse effect on mortality rates and the financial sustainability of a life insurer in 2020 than that in 2021.

**Figure 6:**
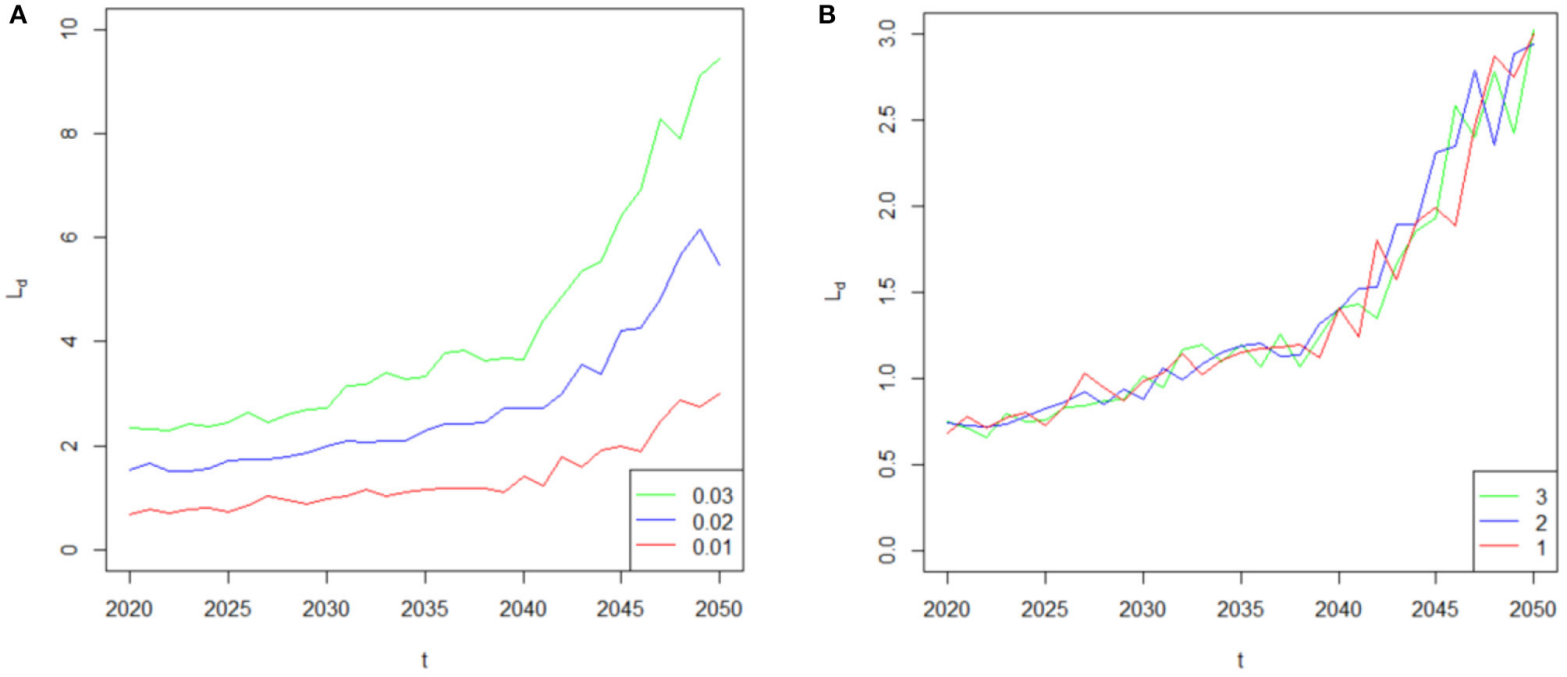
**(A)** Sensitivity of the liability difference to the parameter λ_τ_. **(B)** Sensitivity of the liability difference to the parameter κ.

Given that the COVID-19 induced mortality risk will impose a negative effect on the financial sustainability of life insurer, a natural question that follows is: what is the relative importance of the jump event *j* in determining the liability? Answering this question is very important because it describes the characteristics of the jump event *j* in determining the liability, which directly affects the life insurer's financial sustainability. Therefore, we consider the arrival rate λ_τ_ and the duration κ of the jump event j by varying their assumed values to extend our analysis. The sensitivity analysis results are based on the COVID-19 mortality rates in 2020, which are shown in Figure 6. In Figure 6A, the red curve, blue curve and green curve represent the basic case λ_τ_ = 0.01, λ_τ_ = 0.02 and λ_τ_ = 0.03, respectively. As can be seen from this plot, the value of the liability difference *L*_*d*_ markedly increases with the value of arrival rate λ_τ_. For example, if the pandemic is a two (three)-in-one-hundred year event, λ_τ_ = 0.02 (λ_τ_ = 0.03); that is, the jump event *j* will occur with greater frequency than λ_τ_ = 0.01, and then the maximum value of the liability difference will increase from 3.00 million dollars to 6.16 (9.44) million dollars; thus suggesting that a life insurer's financial sustainability is an decreasing function of the arrival rate λ_τ_. We then retain the assumption that pandemics usually last for short time periods by changing the value of κ from κ = 1 into κ = 2 and κ = 3 to analyze the effect of duration κ on the liability difference *L*_*d*_. However, in Figure 6B, the liability difference *L*_*d*_ is less sensitive to the duration κ of the jump event *j*. This is because the randomness of the Poisson process denominates the value of the liability difference *L*_*d*_. The time of the jump event τ_*j*_ cannot be controlled and may not occur simultaneously for each duration κ, thus having an uncertain effect on the liability difference *L*_*d*_. Therefore, the parameter κ does not have a clear influence on the liability difference *L*_*d*_ and the financial sustainability based on the short-term effect of a pandemic on mortality rates. In general, the arrival rate λ_τ_ of the jump event *j* produces a much more serious effect on the financial sustainability of the life insurer than the short-term duration κ.

## Additional Analysis: Interest Rate Risk

Interest rate risk is indispensable in life insurers' financial sustainability. To model the liability of the life insurer more appropriately, we must include both mortality and interest rate risk. Therefore, we need a model of future interest rates, which involves stochastic simulation. In this section, we assume that the interest rates instead follow a mean-reverting diffusion process in continuous time. With sufficiently strong mean reversion, the interest rates will tend to move toward the long-term mean. For simplicity, the commonly used one-factor Vasicek and CIR models meet our requirements.

### Vasicek Interest Rate Model

Under the Vasicek model, the dynamics of the interest rates can be expressed as follows:


(15) $$\begin{array}{r} dr_{t}=a_{V}\left(b_{V}-r_{t}\right)dt+\sigma_{V}dW_{t}. \end{array}$$

In (15), the interest rate *r*_*t*_ will revert to the long term mean *b*_*V*_ with the reversion speed *a*_*V*_, and random increments will be captured by the standard Brownian motion *dW*_*t*_ with instantaneous volatility σ_*V*_. Following Zhou and Mamon (2012), the closed form solution for each parameter based on the quasi-maximum likelihood estimation is given by (45):


(16) $$\hat{a_{V}}=-\frac{\left(M-1\right)\left(\sum_{i=1}^{M-1}r_{i}r_{i+1}-\sum_{i=1}^{M-1}r_{i}^{2}\right)+\sum_{i=1}^{M-1}r_{i}\left(\sum_{i\;=1}^{M-1}r_{i}-\sum_{i=1}^{M-1}r_{i+1}\right)}{\left[\left(M-1\right){\sum_{i=1}^{M-1}r_{i}^{2}}^{\;}-{\left(\sum_{i=1}^{M-1}r_{i}\right)}^{2}\right]},$$

(17) $$\hat{b_{V}}=-\frac{-\sum_{i=1}^{M-1}r_{i}r_{i+1}\sum_{i\;=1}^{M-1}r_{i}+\sum_{i=1}^{M-1}r_{i+1}\sum_{i=1}^{M-1}r_{i}^{2}}{\sum_{i\;=1}^{M-1}r_{i}\left(\sum_{i\;=1}^{M-1}r_{i}-\sum_{i\;=1}^{M-1}r_{i+1}\right)+\left(N-1^{\;}\right)(\sum_{i=1}^{M-1}r_{i+1}^{\;}r_{i}-\sum_{i=1}^{M-1}r_{i}^{2})},$$

(18) $$\begin{array}{r} \hat{\sigma_{V}}=\sqrt{\frac{\sum_{i=1}^{M-1}{\left(r_{i+1}-r_{i}-\hat{a_{V}}\left(\hat{b_{V}}-r_{i}\right)\right)}^{2}}{\left(M-1\right)}}, \end{array}$$

where *M* is the number of observation*s*. The constant is the difference between *t*_*i*+1_ and *t*_*i*_.

### CIR Interest Rate Model

The disadvantage of the Vasicek model is that the interest rate will drop below zero (46), but only positive interest rates will exist in the CIR model. Therefore, we extend our analysis by considering the CIR interest rate model. The dynamics of the interest rates in the CIR stochastic model are described by the Ornstein-Uhlenbeck stochastic process:


(19) $$\begin{array}{r} dr_{t}=a_{C}\left(b_{C}-r_{t}\right)dt+\sigma_{C}\sqrt{r_{t\;}}d{W_{\;}}_{t}, \end{array}$$

where parameter *a*_*V*_ is the reversion speed. Parameters *b*_*V*_ and σ_*V*_ are the long-term mean and the volatility, respectively. On the basis of the closed from solution of the CIR model from Zhou and Mamon (2012), the parameter estimation can be obtained via the method of quasi-maximum likelihood, which can be expressed as follows (45):


(20) $$\hat{a_{C}}=\frac{\begin{array}{c} -{\left(M-1\right)}^{2}+\left(M-1\right)\sum_{i=1}^{M-1}\frac{r_{i+1}}{r_{i}}\\ +\sum_{i=1}^{M-1}\frac{1}{r_{i}}(\sum_{i\;=1}^{M-1}r_{i}-\sum_{i=1}^{M-1}r_{i+1}) \end{array}}{\left[-{\left(M-1\right)}^{2}+\sum_{i=1}^{M-1}\frac{1}{r_{i}}\sum_{i\;=1}^{M-1}r_{i}\right]},$$

(21) $$\hat{b_{C}}=\frac{\sum_{i=1}^{M-1}\frac{r_{i+1}}{r_{i}}\sum_{i\;=1}^{M-1}r_{i}-\left(M-1\right)\sum_{i=1}^{M-1}r_{i+1}}{\begin{array}{c} -{\left(M-1\right)}^{2}+\left(M-1\right)\sum_{i=1}^{M-1}\frac{r_{i+1}}{r_{i}}\\ +\sum_{i=1}^{M-1}\frac{1}{r_{i}}(\sum_{i\;=1}^{M-1}r_{i}-\sum_{i=1}^{M-1}r_{i+1}) \end{array}},$$

(22) $$\begin{array}{r} \hat{\sigma_{C}}=\sqrt{\frac{\sum_{i=1}^{M-1}{\left(\frac{r_{i+1}-r_{i}-\hat{a_{C}}\left(\hat{b_{C}}-r_{i}\right)}{\sqrt{r_{i}}}\right)}^{2}}{\left(M-1\right)}}. \end{array}$$

### Numerical Calculations

In this section, we use the annualized monthly yield of the 30-year U.S. Stripe Index from February 28, 1985 to December 31, 2019 in the Datastream database to calibrate the Vasicek and CIR models based on equation (16)-(18) and (20)-(22), respectively. The estimated parameters of these two interest rate models are reported in Table 2. Our results show that the reversion speed and long-term mean in these two models are almost the same, but the instantaneous volatility in the Vasicek model (σ_*V*_ = 0.0025) is much smaller than that in the CIR model (σ_*C*_ = 0.0110), which suggests a smoothing change of interest rates in the Vasicek model.

**Table 2:** Quasi-maximum likelihood estimation of Vasicek and CIR models.

**Vasicek**	**Estimate**	**CIR**	**Estimate**
*a*_*v*_	0.0141	*a*_*C*_	0.0138
*b*_*v*_	0.0414	*b*_*C*_	0.0411
σ_*v*_	0.0025	σ_*C*_	0.0110

**Table 3:** Probabilities of negative interest rates under the Vasicek model.

**T**	**Pr <0**	***t***	**Pr <0**
2020	0.0000	2036	0.0011
2021	0.0000	2037	0.0013
2022	0.0000	2038	0.0015
2023	0.0000	2039	0.0016
2024	0.0000	2040	0.0018
2025	0.0000	2041	0.0020
2026	0.0000	2042	0.0022
2027	0.0000	2043	0.0024
2028	0.0001	2044	0.0026
2029	0.0001	2045	0.0028
2030	0.0002	2046	0.0030
2031	0.0003	2047	0.0031
2032	0.0004	2048	0.0033
2033	0.0006	2049	0.0035
2034	0.0007	2050	0.0036
2035	0.0009		

**Figure 7:**
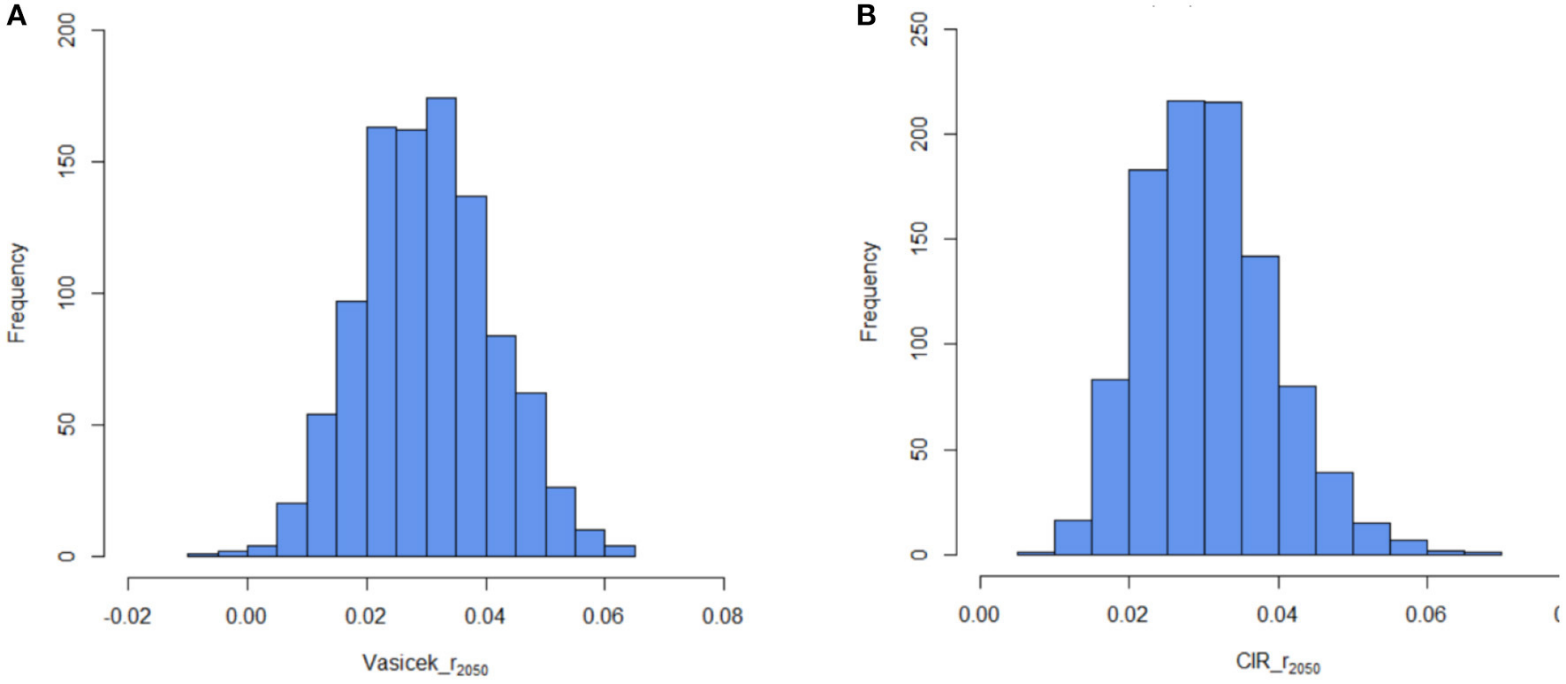
**(A)** Simulation with the interest rate at *t* = 2050 based on the Vasicek model. **(B)** Simulation with the interest rate at *t* = 2050 based on the CIR model.

Then we use the U.S. Stripe Index data at the end of 2019 as the initial interest rate *r*_2019_ = 0.0247 and simulate for 1,000 times from 2020 to 2050 by using the estimated parameters based on the Vasicek and CIR models. Figures 7A,B show the typical distribution of the *r*_2050_ based on the Vasicek model and the CIR model, respectively. In Figure 7, we can observe that both the Vasicek and CIR models have a small number of very low and very high simulated values, although the interest rate process is mean reverting (47). Specifically, the Vasicek model has the disadvantage of allowing negative interest rates as shown on the left side of Figure 7A.

To describe the main drawback of the Vasicek model, we also compute the likelihood of negative rates in the Vasicek model by using the formula:


(23) $$Pr\left(r_{t}<0\right)=\varphi (-\frac{r_{0}e^{-a_{V}t}+b_{V}(1-e^{-a_{V}t})}{\sigma_{V}\sqrt{\frac{1-e^{-2a_{V}t}}{2a_{V}}}})$$

where φ(·) represents the cumulative distribution function of the standard normal. According to the estimated parameters *a*_*V*_ = 0.0141, *b*_*V*_ = 0.0414 and σ_*V*_ = 0.0025, the probabilities of negative interest rates from *t* = 2020 to *t* = 2050 are shown in Table 3. Our results suggest that the probabilities of negative rates are almost negligible at time *t* with the estimated parameters above. For example, the maximum probability of negative rates is only 0.0036, and thus only about 4 interest rates will be less than zero when we take 1000 simulation paths.

**Figure 8:**
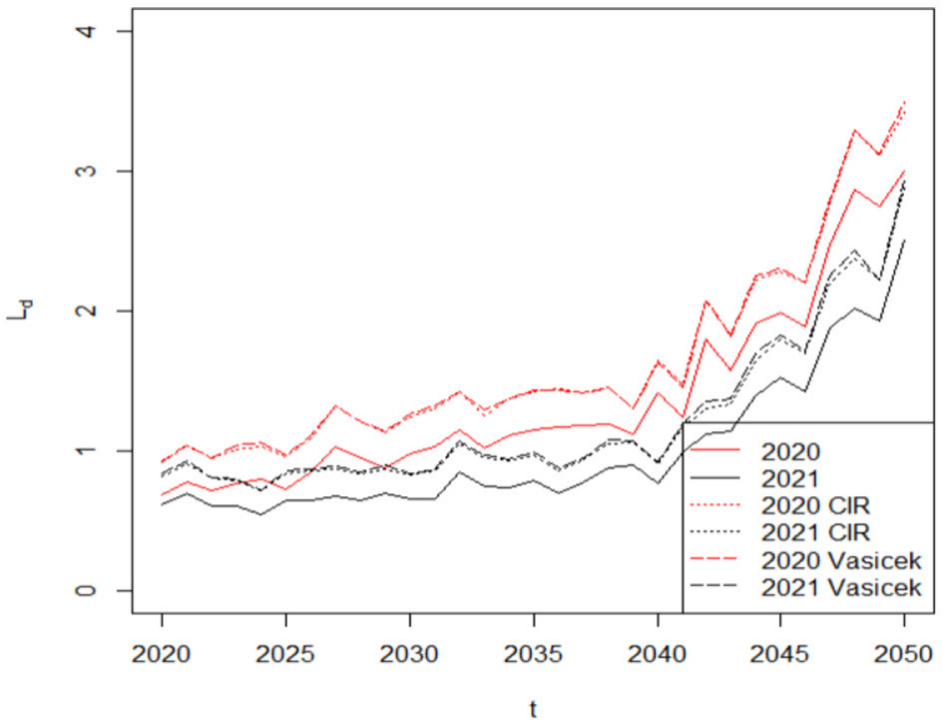
To describe the main drawback of the Vasicek model, we also compute the likelihood of negative rates in the Vasicek model by using the formula: Value of the liability difference at time *t*, including both mortality risk and interest rate risk.

Finally, we use the simulated results based on the Vasicek and CIR models to calculate the liability difference in 2020 and 2021. According to the monthly weighted average table from the DB plan sponsors, the maximum rate that they may be used is 120% of the weighted average of 30-year Treasury securities. Accordingly, the discount rates are assumed to be 1.2 times the interest rates in our study $r_{L,t}=r_{t}^{*}1.2$. Our results based on the CIR and Vasicek models are shown in Figure 8. The red curves in Figure 8 represent the liability difference in 2020, for example. The solid curve, dotted curve and dashed curve show the increasing trend of the liability difference *L*_*d*_ based on the fixed discount rates *r*_*L, t*_ = 0.05, the CIR model and the Vasicek model, respectively. While illustrative results based on stochastic interest rate models are similar due to the nearly identical reversion speed and long-term mean, the value of the liability difference *L*_*d*_ after considering the interest rate risk will increase because the 1.2 times the long term mean is lower than 0.05 (0.04111 × 0.20 = 0.0493, 0.04141 × 0.20 = .0497). Specifically, in Figure 8, the maximum value of the liability difference *L*_*d*_ based on the fixed discount rate *r*_*L, t*_ = 0.05 is 3.00 million dollars, which is smaller than that based on the Vasicek model (3.49 million dollars) and the CIR model (3.42 million dollars). Therefore, the accuracy of the liability difference *L*_*d*_ increases as we incorporate interest rate risk, which should help life insurers better analyze their financial sustainability. Overall, life insurers' financial sustainability deteriorates with declining discount rates.

## Conclusions

In this article, we define a stochastic mortality model with a temporary mortality jump process to capture the effect of COVID-19 on the mortality rates among people at different ages. To show the effect of mortality risk on the life insurer's financial sustainability, we then define the liability of the life insurer to analyze the difference during versus before COVID-19. Finally, we also consider the interest rate risk by adopting the Vasicek and CIR stochastic interest rate models to calculate the liability of the life insurer. Our results show that the liability of the life insurer in the wake of COVID-19 is higher than that in the pre-COVID-19 period because of the lower survival probability caused by this pandemic. Consequently, COVID-19 imposes a negative effect on life insurers' financial sustainability. In addition to liability, COVID-19 has also greatly decreased investment income based on asset, which suggests that we should consider the overall firm risk caused by COVID-19. Consequently, it is fruitful to extend our research to the total surplus setting. We leave this question for future research.

## Data Availability Statement

The original contributions presented in the study are included in the article/Supplementary Material, further inquiries can be directed to the corresponding author.

## Author Contributions

All authors listed have made a substantial, direct and intellectual contribution to the work, and approved it for publication.

## Conflict of Interest

The authors declare that the research was conducted in the absence of any commercial or financial relationships that could be construed as a potential conflict of interest.

## Publisher's Note

All claims expressed in this article are solely those of the authors and do not necessarily represent those of their affiliated organizations, or those of the publisher, the editors and the reviewers. Any product that may be evaluated in this article, or claim that may be made by its manufacturer, is not guaranteed or endorsed by the publisher.
